# High-Pressure Spark Plasma Sintering (HP SPS): A Promising and Reliable Method for Preparing Ti–Al–Si Alloys

**DOI:** 10.3390/ma10050465

**Published:** 2017-04-27

**Authors:** Anna Knaislová, Pavel Novák, Sławomir Cygan, Lucyna Jaworska, Marcello Cabibbo

**Affiliations:** 1 Department of Metals and Corrosion Engineering, University of Chemistry and Technology Prague, Technická 5, 16628 Prague, Czech Republic; panovak@vscht.cz; 2 The Institute of Advanced Manufacturing Technology, Wroclawska 37A, 30-011 Krakow, Poland; slawomir.cygan@ios.krakow.pl (S.C.); lucyna.jaworska@ios.krakow.pl (L.J.); 3 DIISM/Università Politecnica delle Marche, Via Brecce Bianche, 60131 Ancona, Italy; marcello.cabibbo@univpm.it

**Keywords:** high-pressure spark plasma sintering (HP SPS), powder metallurgy (PM), intermetallics, hardness

## Abstract

Ti–Al–Si alloys are prospective material for high-temperature applications. Due to low density, good mechanical properties, and oxidation resistance, these intermetallic alloys can be used in the aerospace and automobile industries. Ti–Al–Si alloys were prepared by powder metallurgy using reactive sintering, milling, and spark plasma sintering. One of the novel SPS techniques is high-pressure spark plasma sintering (HP SPS), which was tested in this work and applied to a Ti–10Al–20Si intermetallic alloy using a pressure of 6 GPa and temperatures ranging from 1318 K (1045 °C) to 1597 K (1324 °C). The low-porosity consolidated samples consist of Ti_5_Si_3_ silicides in an aluminide (TiAl) matrix. The hardness varied between 720 and 892 HV 5.

## 1. Introduction

The high-pressure spark plasma sintering (HP SPS) is an innovative variant of a well-established spark plasma sintering method for the sintering of materials, especially superhard materials based on polycrystalline diamond or cubic boron nitride, ceramic composites, nanopowders, or refractory materials. Using this and other new SPS methods, it is also possible to synthetize materials with metastable phases or intermetallic alloys [1,2,3,4]. The HP SPS method belongs to a new generation of SPS processes, and it was already successfully employed for example for the sintering of ZrC-based composites [5]. Among these processes, pulsed electric current SPS [1], hot pressing before SPS, which involves high-temperature exposures and long-term durations [2], and self-propagating high-temperature sintering prior to SPS [3] are now well-established modern and reliable SPS techniques. The pulsed electric current SPS has the advantage of heating the compacting system from outside and inside, which guarantees quite favorable powder densification [4]. The HP SPS is generally carried out under ultra-high pressure (up to 8 GPa) for a short time. Pressure facilitates the new arrangement of grains, reduces the diffusion during the sintering of the material, increases the material density, and eliminates porosity, thus reducing the temperature and sintering duration. The short sintering time (up to 3 min) decreases the major drawback potential induced by the high-temperature process, which is the grain growth tendency with temperature and time in the sintered material [6,7]. Moreover, due to the high pressure of the process, it can be expected that the process can be applied for intermetallics similarly as for the minerals [8]. Initially, brittle materials after the process will be characterized by improved mechanical properties, due to the extended plastic deformation range. 

**Figure 1:**
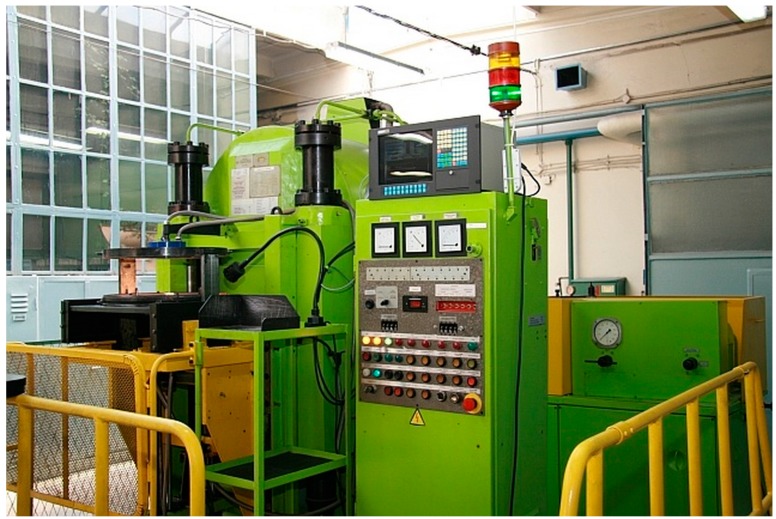
High-pressure spark plasma sintering (HP SPS) apparatus in the Institute of Advanced Manufacturing Technology, Krakow.

The HP SPS apparatus (Figure 1) consists of a high tonnage hydraulic press equipped with Bridgman type anvil and a generator of direct-pulsed current. The entire process of sintering is managed by a computer control system. The Bridgman type anvil has a toroidal shape, which helps to achieve the quasi-isostatic compression on the material due to the plastic deformation of the gasket with sintered material. The heating is carried out by a 1 kHz pulsed current that passes directly through the graphite heater in the gasket and through conductive sintered material as well. This method of heating, compared to conventional sintering methods, has the main advantage of a lower sintering temperature. Another advantage given by direct-pulsed current heating is derived from the application of very high heating and cooling rates and the surface activation of powders by in situ plasma cleaning, which can lead to the synthetizing of new phases [9].

The techniques of sintering and preparation of intermetallic compounds is quite a challenging task. For this reason, conventional melting metallurgy has been the most common preparation method of intermetallic compounds yet. Nowadays, due to the high melting points of intermediary phases and the exothermic reaction during their formation, new methods of preparation are currently under investigation and promising results started to appear in international journals. In this sense, powder metallurgy may be one of them. It is the rapidly growing technology dealing with the production of powders and then their consolidation. Intermetallic compounds can be produced by reactive sintering, but the high porosity of the resulting samples complicates their use in many branches of industry. The step of spark plasma sintering (SPS) after reactive sintering can be the appropriate way to achieve compacted materials virtually without porosity (or bearing a minimal and almost insignificant porosity level) and with good mechanical properties. The HP SPS has been shown to successfully produce intermetallic compounds [10]. A further issue consists of the porosity level of the produced material, which can be minimized by applying high pressure during the sintering process [11,12,13]. 

Ti–Al–Si alloys could be a substitute of conventional iron- and nickel-based heat resistant alloys for high-temperature environments, which contain also chromium and other critical raw materials (CRM) [14,15]. One of the key mechanical properties of the Ti–Al–Si system is the particularly high wear resistance shown. Therefore, it can be beneficial to consider this alloy as a CRM-free (or low-CRM) material for high-temperature applications and in principle as a tool material. High brittleness at room temperature is the main drawback of the Ti–Al–Si alloy system, which is likely to be solved by modifying the resulting structure through an appropriate and dedicated, processing technology [16,17].

## 2. Materials and Methods

Ti–10Al–20Si alloy was prepared by powder metallurgy using self-propagating high-temperature synthesis (SHS), milling, and consolidation by high-temperature, high-pressure spark plasma sintering in IZTW Krakow. The main problem of the material produced by SHS method itself is a porous and often very heterogeneous structure. For these reasons, we included milling in a vibratory mill after SHS and HP SPS for subsequent consolidation of the milled powder. It was aimed at a formation of a more homogeneous structure with lower porosity. 

SHS of pre-pressed powders Ti–10Al–20Si alloy was conducted for 30 min in electric resistance furnace preheated to a temperature of 1173 K (900 °C). After the SHS process, the ampoules with samples were air-cooled to laboratory temperature. Prepared samples were then milled in vibrating laboratory mill VM4 (Brio Hranice, Czech Republic) for 7 min.

**Figure 2:**
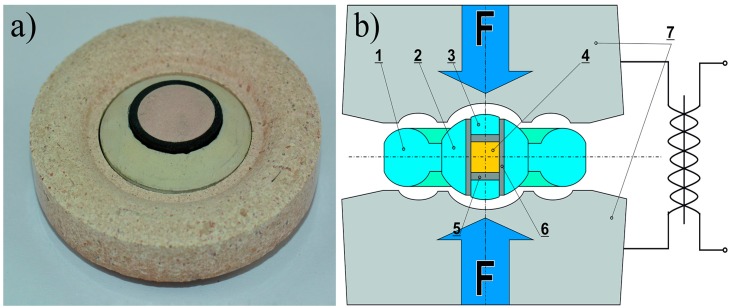
View of the high-pressure gasket assembly (**a**); cross-section diagram of the sintering process (**b**): 1—ceramic gasket (outer part); 2—ceramic gasket (inner part); 3—ceramic disc; 4—sample; 5—graphite disc; 6—graphite tube; 7—sintered carbide dies.

In IZTW Krakow, powders were pre-pressed at 100 MPa into discs 15 mm in diameter and 5 mm high. These green bodies were then put in the graphite heater, which was subsequently inserted into remaining ceramic elements of the high-pressure spark plasma sintering gasket assembly. Sintering was carried out at the pressure of 6 GPa. The temperatures were chosen from 1318 K (1045 °C) to 1597 K (1324 °C). These temperatures were adjusted by calibration for the 40 ms impulse length and the 20 ms impulse interval, where temperature changed with the change of the impulse degree of filling. The degree of filling is a programmable parameter of the HP SPS device, which describes the percentage of filling of the programmed 40 ms impulse of 1 kHz direct pulsed current with initial 1 ms pulses. Temperature calibration is required due to the lack of possibility to measure temperature directly during sintering processes. The duration of the sintering was from 30 to 150 s depending on the sintering temperature. Figure 2 shows the prepressed tablet in the form. In Table 1, the conditions of HP SPS are summarized.

This entire experimental work was aimed at a formation of a more homogeneous structure with lower porosity. For this reason, the appropriate time of heating given by experiences of HP SPS operators in Krakow was used. At higher temperatures, compared to lower temperatures, it was not possible to use the same time because there was a higher risk of explosions and damage to the equipment. This is due to the fact that these materials required a lower pressure range (6 GPa instead of the maximum 8 GPa) to be obtained with gasket assembly in order to limit the presence of cracks. When employing a lower range of pressure for this gasket assembly, the applicable temperature and/or the process time also decrease (the higher the temperature, the shorter the possible sintering duration). 

The reference material was prepared from the same powder by conventional spark plasma sintering at the temperature of 1373 K (1100 °C) for 5 min using the heating rate of 100 K/min and the pressure of 48 MPa in UCT Prague using the FCT HP D10 SPS device (FCT Systeme GmbH, Rauenstein, Germany).

The microstructure of the Ti–Al–Si alloys was observed by the metallographic optical microscope Olympus PME3 and was documented using the digital camera Carl Zeiss AxioCam ICc3 (Carl Zeiss, Jena, Germany) and AxioVision software (version 4.8.2, Carl Zeiss, Jena, Germany). Porosity and pore size were evaluated by using image analyzer Lucia 4.8. (Laboratory Imaging, Czech Republic) The electron microscope TESCAN VEGA 3 LMU equipped with an EDS analyzer (SEM-EDS) (SEM: Tescan, Brno, Czech Republic, EDS: Oxford Instruments, High Wycombe, Great Britain) was used for a more detailed view of the structure and an identification of present phases. Vickers hardness with a load of 5 kg (HV 5) was measured from 10 indentions into the polished compacted sample. 

## 3. Results and Discussion

**Figure 3:**
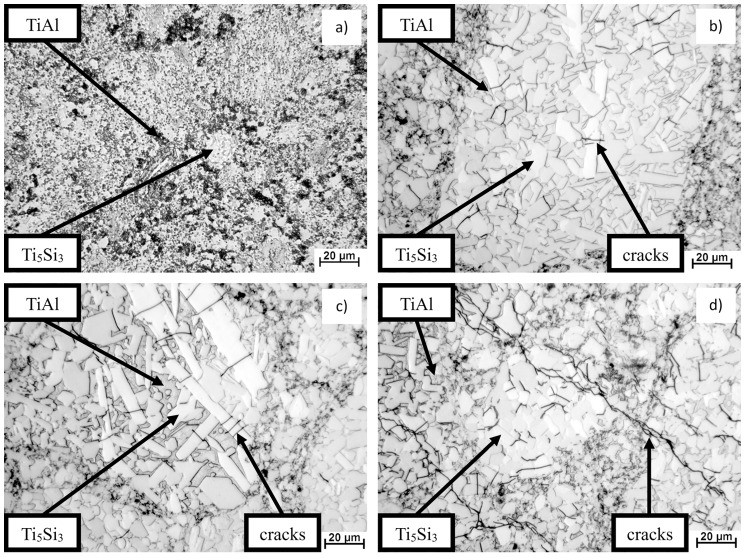
Microstructure of Ti–10Al–20Si (**a**) prepared by SPS at 48 MPa and 1373 K (1100 °C); (**b**) prepared by HP SPS at 6 GPa and 1318 K (1045 °C); (**c**) 1422 K (1149 °C); (**d**) 1597 K (1324 °C).

In Figure 3, the microstructure of the Ti–10Al–20Si alloy sintered at various temperatures and pressures is shown. The samples were etched by Kroll’s reagent (10 mL of HF + 5 mL of HNO_3_ + 50 mL of H_2_O). The four prepared compacts showed a very heterogeneous microstructure characterized by fine sharp-edged Ti_5_Si_3_ silicides embedded in a TiAl matrix. The detected phase composition was independent from the sintering temperature and applied pressure. In particular, the microstructure of Figure 2b–d clearly showed cracks through the structure. This is likely to be generated by the high pressure of sintering. On these samples, it is possible to see many microcracks in the silicides, which are believed to have been initiated and propagated by the thermal expansion of the sharp-edged Ti_5_Si_3_ silicides (essentially because of their morphology).

**Figure 4:**
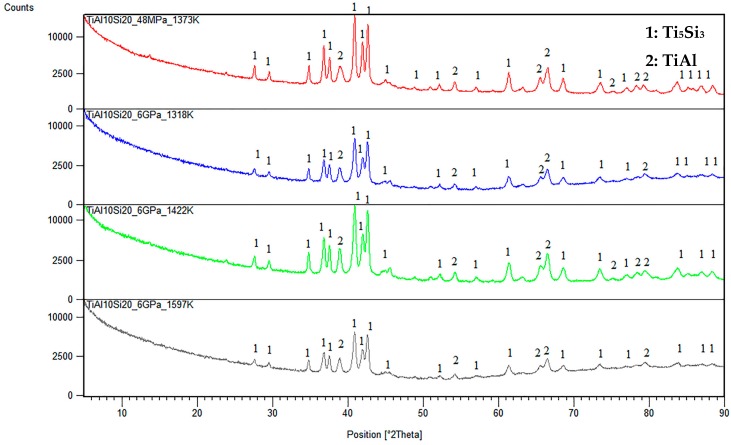
XRD patterns of powders after milling and compacted alloys prepared by SPS. 1: Ti_5_Si_3_; 2: TiAl.

Phase composition is shown in Figure 4. The phase composition of milled powder and after compaction is equal. The TiAl10Si20 alloy is characterized by Ti_5_Si_3_ silicides and the TiAl matrix. The formation of silicide Ti_5_Si_3_ is given by the high mutual affinity of titanium and silicon and its high thermodynamic stability.

**Figure 5:**
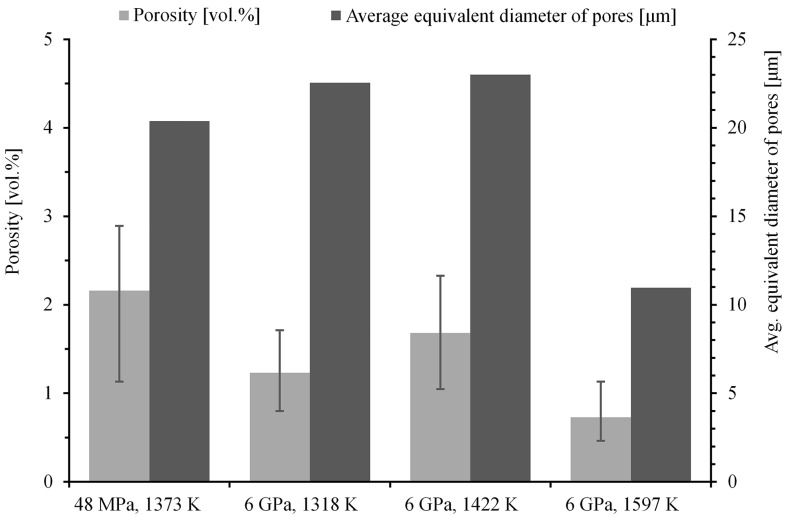
Porosity and pore size of the Ti–10Al–20Si alloy prepared by SPS and HP SPS at different temperatures.

The porosity and pore size of Ti–10Al–20Si alloys is reported by the histogram in Figure 5. It shows that the high pressure induced a significant overall alloy porosity reduction and higher densification. Thus, the Ti–10Al–20Si alloy sintered at 1597 K (1324 °C) showed the lowest porosity level accounting for less than 1%, corresponding to the lowest mean pore size of 11 µm (in equivalent diameter calculated as $d=\sqrt{\frac{4\pi }{A}}$ where A is the measured pore area). It is interesting to note that the mean pore size did not change significantly with increasing sintering temperature in the range of 1373–1422 K (1100–1149 °C), while, in this temperature range, the porosity volume fraction varied in the range from 1.2 to 2.2%. These results show that a proper alloy densification can be obtained for temperatures not below ~1597 K (~1324 °C) at 6 GPa.

**Figure 6:**
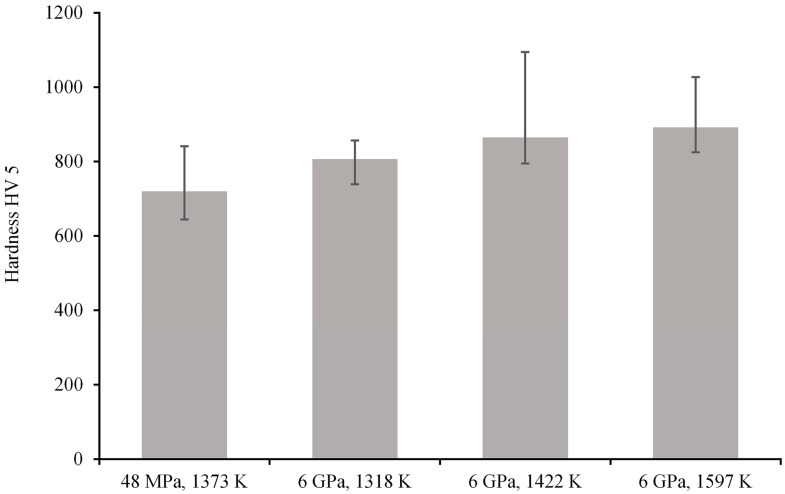
Hardness of the Ti–10Al–20Si alloy prepared by SPS and HP SPS at various temperatures.

The hardness measurements, performed on three different HP SPS Ti–10Al–20Si intermetallic alloy samples, varied from 720 to 892 HV 5 (Figure 6). For a sake of comparison, the hardness value of the same alloy prepared under a pressure of 48 MPa and using a conventional SPS at UCT Prague is also reported by the histograms in Figure 5. All three alloys prepared by HP SPS had a greater hardness than the conventional SPS Ti–10Al–20Si alloy. In particular, the hardness reached a maximum at HP SPS temperature of 1597 K (1324 °C), so the most properly compacted intermetallic alloy exhibited the hardness of 892 HV. These results show an improvement when compared with previously published data on similar binary intermetallic alloys (Ti–Al) produced by conventional SPS at various temperatures up to 1373 K (1100 °C) [18]. 

## 4. Conclusions

The Ti–Al–Si alloys were successfully prepared by the HP SPS method in the Institute of Advanced Manufacturing Technology in Krakow. This method has shown favorable potentials in the case of preparation of intermetallic alloys of the Ti–Al–Si system (namely, a Ti–10Al–20Si alloy was produced). The resulting structure of the alloys exhibits low porosity (up to 1.5 vol %) with a relatively small size of pores, but we can often see cracks through the structure and in the silicides, which can be caused by a high pressure of sintering and/or relatively quick cooling after SPS. In particular, the maximum temperature of 1597 K (1324 °C) at 6 GPa of the HP SPS process was able to show an alloy porosity lower than 1 vol % with a mean pore size of ~10 µm and a quite high hardness HV5 of approximately 900. Therefore, for all those applications, where high hardness is necessary, the high-pressure SPS sintering seems to be a promising and reliable new SPS technique.

## Figures and Tables

**Table 1:** Conditions of sintering in HP SPS.

Sample		Pressure (GPa)	Time (s)	Degree of Filling (%)	Temperature (K)
Ti–10Al–20Si	#1	6 ± 0.2	150	55	1320 ± 50
Ti–10Al–20Si	#2	6 ± 0.2	130	57	1420 ± 50
Ti–10Al–20Si	#3	6 ± 0.2	30	60	1600 ± 50
